# The Impact of Demographic Factors on Adults’ Use of Artificial Intelligence for Seeking Nutritional Health Information in Saudi Arabia

**DOI:** 10.3390/healthcare14213702

**Published:** 2026-11-01

**Authors:** Rehab Aldahash, Reem Alzubaidi, Farah Alawad, Lulu Alanazi, Amal Alanazi

**Affiliations:** Department of Health Sciences, College of Health and Rehabilitation Sciences, Princess Nourah Bint Abdulrahman University, P.O. Box 84428, Riyadh 11671, Saudi Arabia; 444001301@pnu.edu.sa (R.A.); 444001288@pnu.edu.sa (F.A.); 444001297@pnu.edu.sa (L.A.); 444000729@pnu.edu.sa (A.A.)

**Keywords:** artificial intelligence, nutritional health information, digital health literacy, technology acceptance model, cross-sectional study, multivariate analysis, Saudi Arabia

## Abstract

**Highlights:**

**What are the main findings?**

- The prevalence of AI use for seeking nutritional health information was high, with ChatGPT being the most frequently used tool.
- Higher AI use was observed among females, young adults, single individuals, and those without chronic diseases.

**What are the implications of the main findings?**

- AI use was associated with reduced visits for minor health issues, greater acceptance of AI-based diagnostics, and improved decision-making.
- The findings support strengthening the integration of AI with Ministry of Health platforms to improve access to reliable nutritional health information.

**Abstract:**

Background: Driven by Saudi Vision 2030 and widespread internet access, artificial intelligence (AI) is increasingly used for nutritional guidance in Saudi Arabia. Despite benefits such as personalized dietary advice, challenges remain, and usage patterns vary across sociodemographic groups. This study investigates the effect of sociodemographic factors on the use of AI for obtaining nutritional health information among adults residing in Saudi Arabia. Methodology: A cross-sectional study was conducted among 446 adult participants aged 18–64 years residing in Saudi Arabia. Data were collected via an online questionnaire from December 2025 and March 2026, covering sociodemographic characteristics, AI use, attitudes, and practices related to nutritional health information. Statistical analysis included chi-square tests and multivariable linear and logistic regression. Results: A total of 380 participants were AI users for nutritional health information, with ChatGPT identified as the most commonly used tool. AI use was significantly higher among females, younger adults, single individuals, and those without chronic disease (*p* < 0.05 for all). After adjustment for demographic confounders, age remained the only independent predictor of frequent AI use, with significantly lower odds observed among adults aged 41 and older (*p* < 0.05). Attitude scores were the strongest independent predictor of nutritional practice (adjusted β = 5.32 per standard-deviation increase, *p* < 0.001), outweighing sociodemographic factors once entered jointly. Reliance on AI was also significantly associated with nutritional practice scores, including reduced dietitian visits for minor issues (*p* = 0.0002), acceptance of AI-based diagnostic suggestions (*p* = 0.0002), and improved dietary decision-making (*p* = 0.0164). Conclusions: AI use for nutritional information is common among a predominantly young, female, single, urban (Central-region), and highly educated sample of Saudi adults, and is shaped more by attitudinal beliefs than by demographic characteristics alone. AI is mainly used for information support rather than final health decisions, serving as a complementary tool in nutrition care.

## 1. Introduction

Digital technologies have substantially changed how individuals seek and obtain health information, and artificial intelligence (AI) has become increasingly integrated into this environment [1,2]. AI is a broad field encompassing computational systems that perform tasks normally requiring aspects of human intelligence, whereas generative AI (GenAI) is a specific category of techniques that generate new content, such as text, images, audio, or video, from patterns learned from data [3]. Within this field, large language models (LLMs) are particularly relevant to consumer health-information seeking. They process natural-language prompts and generate conversational responses tailored to the user’s query, rather than simply directing users to existing sources. ChatGPT is a prominent LLM-based chatbot, and similar conversational platforms have since become widely available [4,5].

Accordingly, in the present study, “AI use” refers specifically to the use of publicly accessible generative-AI and conversational chatbot platforms to seek, obtain, interpret, or generate nutritional health information. This includes the platforms reported in the questionnaire, such as ChatGPT, Google Gemini, Claude, DeepSeek, and Microsoft Copilot, and other comparable tools. The definition therefore excludes other healthcare applications of AI, such as medical imaging, predictive modelling, automated diagnosis, clinical decision-support systems, and administrative data analytics, which are outside the scope of this research. Conversational AI is increasingly of interest for nutrition-related questions, including dietary advice, meal planning, nutrient estimation, and the nutritional management of chronic conditions [6,7,8]. However, the quality of the information it generates is variable. Studies of ChatGPT and other chatbots have identified limitations in the accuracy, completeness, consistency, and nutritional adequacy of generated advice, particularly in complex dietary or clinical situations [6,7,8,9]. Using GenAI for nutritional information should therefore not be equated with access to verified professional advice.

Because output quality varies, the adequacy of AI-based health guidance depends heavily on users’ digital health literacy: the ability to seek, understand, appraise, and apply health information obtained through digital technologies [10] (Norman & Skinner, 2006). Digital health literacy is increasingly recognized as a determinant of health in its own right, shaping who benefits from digital tools and who is left behind. A recent four-country cross-sectional study found significant variation in digital health literacy by age and health status, even in high-income settings with near-universal internet access [11]. Appraisal skills are especially important for generative tools because of their propensity to produce hallucinations, that is, plausible-sounding but factually incorrect or fabricated content. Even in evidence-based tasks, one comparative analysis found hallucination rates of 39.6% for GPT-3.5 and 28.6% for GPT-4 when generating scientific references [12]. When lay users seek nutritional or medical guidance rather than citations, the risk shifts from fabricated references to inaccurate health claims presented with high apparent confidence, and users have no inherent means of detecting the error. This is particularly relevant here, given that a large majority of participants reported using AI tools such as ChatGPT for nutritional health information.

AI-based nutrition tools also raise concerns about algorithmic bias. Reviews of AI applications in dietetics highlight that models are frequently trained on datasets lacking demographic, geographic, and cultural diversity, which risks perpetuating health disparities and producing recommendations poorly suited to underrepresented populations [13]. This concern is not merely theoretical: even beyond generative tools, food-image classification systems trained predominantly on Western food databases show accuracy reductions of 25–30% when applied to non-Western cuisines [14]. This is directly relevant to Saudi and broader Middle Eastern dietary patterns, which remain underrepresented in most publicly available nutrition-AI training datasets. Such bias may affect not only the accuracy but also the cultural appropriateness of AI-generated nutritional advice for the population studied here. Taken together, limited digital health literacy, hallucination risk, and algorithmic bias are three interlinked, globally documented challenges that contextualize, and help explain, the patterns of AI reliance, trust, and demographic variation examined in this study.

The widespread availability of digital technologies provides an important context for understanding the use of generative AI for health and nutrition information. Internet access in Saudi Arabia is now nearly universal, reaching 99% of the population in 2024, while 21.5% of internet users reported using AI tools [15]. However, high levels of connectivity do not necessarily translate into equivalent abilities to find, understand, appraise, and use health information. A large Saudi study of more than 2000 adults found that although 63% of participants demonstrated proficiency in using search engines to obtain health information, 37% experienced difficulty formulating precise health-related queries, and digital health literacy varied significantly according to age and gender [16]. These findings are particularly relevant to generative AI because interacting effectively with conversational systems requires users not only to access the technology but also to formulate appropriate prompts and critically evaluate the information generated. Evidence from Saudi Arabia also indicates that online health-information seeking is associated with sociodemographic characteristics. Previous studies have reported differences according to age, gender, educational level, and socioeconomic characteristics, with younger age, female gender, higher education, and higher income being associated with greater online health-information-seeking behavior in specific populations [16,17]. Thus, near-universal internet access should not be interpreted as evidence of uniform patterns of digital health-information use.

This distinction is increasingly important as generative AI becomes more visible in the Saudi digital environment. A national survey of 1,084 Saudi adults reported that 49% used AI technologies, with ChatGPT being the most frequently used tool (41%), followed by Google Gemini (23%) [18]. These figures describe general AI use rather than specifically the use of generative AI for nutritional health information. They therefore cannot be directly interpreted as estimates of AI-based nutrition-information seeking. Nevertheless, they indicate a substantial level of public exposure to the types of conversational generative-AI platforms examined in the present study. At the same time, the use of AI-generated health information raises important concerns regarding the reliability, completeness, consistency, and appropriateness of generated responses. Studies evaluating generative-AI chatbots in nutrition have demonstrated that these systems can provide useful dietary information but may also produce inaccurate or incomplete nutrient estimates, inappropriate recommendations, or inconsistent responses, particularly when dietary requirements are complex or clinically specific [6,7,8,9]. These limitations reinforce the importance of users’ ability to critically assess AI-generated nutritional information and of appropriate professional oversight when advice concerns disease management or other complex nutritional needs.

The relevance of these issues is consistent with Saudi Arabia’s broader digital transformation agenda under Saudi Vision 2030, which emphasizes the development of digital infrastructure, digital services, and human capabilities [19]. Understanding who uses generative AI for nutritional health information, how frequently these tools are used, and whether use differs across demographic groups can therefore provide context for future digital health-literacy initiatives and responsible-use guidance. Against this background, the present study focuses specifically on generative-AI use for obtaining nutritional health information among adults in Saudi Arabia. The study examines demographic characteristics, including age, gender, educational level, region, socioeconomic status, health status, social status, and body mass index (BMI), in relation to generative-AI use and reliance on these tools for nutritional health information.

Despite the increasing availability and use of generative AI, empirical evidence on how adults use these platforms specifically to obtain nutritional health information remains limited. Existing research has predominantly examined general AI adoption and technology acceptance or evaluated the technical performance and quality of AI-generated health and nutrition content. Less is known about the characteristics of adults who use generative AI as a source of nutritional health information, the extent to which they rely on these tools, and whether patterns of use differ according to sociodemographic and health-related characteristics. Furthermore, evidence from Saudi Arabia has primarily addressed online health-information seeking and digital health literacy more broadly, while the use of conversational generative-AI platforms for nutritional information has received comparatively little attention [16,17]. Addressing this gap is particularly relevant in a setting characterized by near-universal internet access and rapidly increasing exposure to generative-AI technologies. Therefore, this study examined the use of generative AI for obtaining nutritional health information among adults in Saudi Arabia. Specifically, it aimed to: (1) describe patterns of generative-AI use for nutritional health information; (2) examine sociodemographic and health-related factors associated with generative-AI use and reliance on these tools; and (3) assess the relationship between attitudes toward generative AI and nutrition-related practices.

Beyond describing the prevalence and correlates of generative-AI use, adoption of these tools for health information can be understood through established technology-acceptance theory. The Technology Acceptance Model (TAM), originally proposed by Davis (1989) [20], posits that perceived usefulness and perceived ease of use are fundamental determinants of users’ acceptance and subsequent use of a technology. Subsequent applications of TAM in health-information technologies have demonstrated the relevance of these constructs for understanding consumers’ acceptance of digital health technologies, while also identifying additional factors—including trust—as important determinants of technology acceptance [21,22]. This extension is particularly relevant to generative AI used for health information because users must decide not only whether these systems are useful and easy to interact with, but also whether the information they generate is sufficiently reliable and trustworthy to inform their decisions. A systematic review of AI acceptance research found that extended TAM was among the most frequently used theoretical approaches and that perceived usefulness, trust, attitudes, and effort expectancy were consistently associated with behavioral intention, willingness to use, or use behavior across AI applications [23]. Accordingly, the present study adopts a trust-extended TAM perspective, in which sociodemographic characteristics are considered external factors, perceived usefulness and perceived ease of use represent core technology-acceptance beliefs, trust represents an additional belief relevant to reliance on generative-AI outputs, and self-reported reliance and nutrition-related practices represent behavioral outcomes. This framework provides a structured basis for examining whether demographic characteristics and users’ perceptions of generative AI are associated with how adults use and rely on these tools for nutritional health information.

## 2. Materials and Methods

### 2.1. Study Design and Subjects

This cross-sectional study was conducted among adults aged 18–64 years residing in Saudi Arabia. Data were collected between December 2025 and March 2026 using a structured online questionnaire. The questionnaire assessed participants’ use of generative AI platforms, including ChatGPT/GPT, Google Gemini (formerly Bard AI), Microsoft Copilot (formerly Bing Chat), and other generative AI tools reported by respondents (e.g., Claude and DeepSeek), to obtain nutritional health information. Consistent with the definition given in the Introduction, “AI use” refers to the use of publicly accessible generative AI and conversational chatbot platforms to seek, obtain, interpret, or generate nutritional health information.

The study was conducted in accordance with the Declaration of Helsinki and approved by the Institutional Review Board of Princess Nourah bint Abdulrahman University (Approval No. 25-0705, dated [27 November 2025]). Electronic informed consent was obtained before participants accessed the questionnaire, and the study purpose and objectives were explained at that point. Participation was entirely voluntary. Participants were informed of their right to withdraw at any stage, and a contact method was provided for inquiries. No personally identifiable information was collected, and all responses were anonymized and numerically coded to ensure confidentiality.

### 2.2. Study Sample and Sampling Technique

A non-probability convenience sampling approach was used. The target population comprised Saudi and non-Saudi adults aged 18–64 years living in Saudi Arabia. Participation was voluntary and based on self-selection. Participants were recruited through social media channels (WhatsApp, X, Telegram, Instagram, and LinkedIn) and at selected public and healthcare/educational locations (Riyadh Metro, Princess Nourah bint Abdulrahman University, a nutrition clinic, and a primary healthcare center). Eligible respondents were adults within this age range who resided in Saudi Arabia, provided informed consent, and completed the questionnaire.

The minimum sample size was calculated using the formula for estimating a single proportion:

n = (Z^2^ × P × (1 − P))/E^2^

where:

- P = 0.5 P = 0.5 P = 0.5 (assumed prevalence of 50%);
- Z = 1.96 Z = 1.96 Z = 1.96 (corresponding to a 95% confidence level);
- E = 0.05 E = 0.05 E = 0.05 (margin of error).

Substituting these values:

n = (1.96)^2^ × 0.5 × (1 − 0.5)/(0.05)^2^ ≈ 384

Therefore, the minimum required sample size was 384. Because the survey link was disseminated through social media and QR codes rather than through direct invitations or a predefined sampling frame, the researchers could not determine how many individuals were exposed to the invitation, declined participation, or opened the survey without submitting a response. A response rate therefore could not be calculated, and only submitted entries were available for eligibility assessment and analysis. In total, 512 survey entries were recorded. Of these, 51 respondents did not consent (the questionnaire closed automatically for non-consenting individuals), and 15 entries with missing or incorrect data were identified during data cleaning and excluded. The final analytical sample comprised 439 valid responses, exceeding the minimum required sample size. Of these, 380 participants reported using AI for nutritional health information, and 59 reported not using it.

### 2.3. Data Collection

Data were collected using a structured, self-administered questionnaire comprising four sections: (1) sociodemographic and health characteristics (e.g., nationality, gender, age, marital status, region, education, household income, health insurance, chronic disease status, and BMI); (2) AI usage patterns (whether AI is used, frequency of use, and platforms used); (3) attitudes toward AI in nutritional health (items 15–29) and (4) practices regarding AI-generated advice (items 30–43) (Supplementary File S1).

The Arabic version was used for data collection. Attitudes and practices were measured using Likert-type scales. Attitude and practice items were numerically coded so that higher scores indicate greater reliance on AI. The 14 practice items (items 30–43) were rated on a 5-point frequency scale (1 = Never to 5 = Always). Negatively worded items were reverse-coded, and responses were summed to a total practice score (theoretical range 14–70). A total attitude score was computed from the attitude items.

#### 2.3.1. Theoretical Framework

To move beyond a purely descriptive account of AI adoption, the four sections of the questionnaire were mapped, post hoc, onto the constructs of a trust-extended Technology Acceptance Model (TAM) [15,16,20] (Table 1). Sociodemographic characteristics (Section 1) were treated as external variables that act on use indirectly through beliefs about AI, consistent with the original TAM formulation. Items reflecting confidence in AI’s accuracy and safety (items 16, 17, and 26) were classified as Trust. Items reflecting the perceived value of AI for nutritional and lifestyle outcomes (items 19, 20, 22, 25, and 27–29) were classified as Perceived Usefulness. Items reflecting willingness to depend on AI going forward (items 15, 18, 21, 23, and 24) were classified as Behavioral Intention/Reliance. The practice section (items 30–43), capturing self-reported frequency of AI use and substitution for professional or informal sources, was treated as the Use Behavior outcome. This mapping was developed by the authors as a post hoc theoretical lens applied to an already-validated instrument, rather than a framework used prospectively to design the questionnaire.

#### 2.3.2. Validity and Reliability

Content validity was established through independent review by five health education professionals, whose feedback was incorporated to improve item clarity before data collection. The questionnaire was translated from Arabic into English and back-translated into Arabic by an independent translator to ensure linguistic, conceptual, and cultural equivalence. The translated version was reviewed by experts, and final revisions were completed before participant recruitment, following the forward–back translation and expert-review approach recommended for cross-cultural adaptation of instruments [24,25]. Item-level correlations further supported validity (r > 0.3, *p* < 0.05), exceeding the commonly used minimum of 0.30 for acceptable item-total correlations [26]. Internal consistency was assessed using Cronbach’s alpha (acceptable threshold ≥ 0.70). The Arabic version showed excellent reliability, with α = 0.9256 for the attitudes section and α = 0.9102 for the practices section on the use of AI to obtain nutritional health information [27].

The combination of expert-panel content/face validity [28], item-level correlations [26], and internal consistency (α ≥ 0.70) [27] was considered adequate for the purposes of this study. Formal exploratory or confirmatory factor analysis (EFA/CFA) is primarily indicated when a study aims to develop and validate a new standardized psychometric instrument for repeated use across populations [29]. The present questionnaire, by contrast, was purpose-built to address the specific, pre-defined research questions of this cross-sectional study rather than to establish a reusable scale. This approach mirrors recently published, closely comparable cross-sectional studies of AI knowledge, attitudes, and practice in health-related populations, which similarly relied on expert content review and Cronbach’s alpha, without EFA/CFA, to support instrument validity [30,31]. One of them used the same combination of internal consistency and inter-domain correlations as evidence of construct validity [31]. Nevertheless, Cronbach’s alpha reflects internal consistency rather than dimensionality [32], and the factorial structure of the questionnaire was not tested.

#### 2.3.3. Statistical Analysis

The data were organized and cleaned using JMP software. All statistical analyses were conducted using JMP software, Version 19 (SAS Institute Inc., Cary, NC, USA). Descriptive statistics were used to summarize sociodemographic characteristics and the prevalence of AI use. Categorical variables were presented as frequencies and percentages.

#### 2.3.4. Descriptive and Bivariate Analyses

Descriptive statistics summarized sociodemographic characteristics of AI users in the total sample (n = 380). Categorical variables are presented as frequencies and percentages, and continuous variables (total practice and attitude scores) as mean and standard deviation (SD) and range. Among AI users (n = 380), Pearson’s chi-square tests were used to examine associations between sociodemographic and health characteristics and the frequency of AI use (daily, several times per week, several times per month, once a month). These characteristics were nationality, gender, age group, marital status, education, region, household income, health insurance, BMI category, and each reported chronic condition. Chi-square tests were also used to examine associations of age, gender, and marital status with the perceived benefits of AI for a healthy lifestyle (nutritional habits, physical activity, and exercise planning). Where more than 20% of cells in a contingency table had an expected count below 5, the chi-square approximation was considered unreliable and Fisher’s exact test was used instead. Because these bivariate tests were exploratory, no adjustment for multiple comparisons was applied.

#### 2.3.5. Multivariable Analyses

Attitudes, reliance, and nutritional practice: A multiple linear regression was fitted with total practice score as the dependent variable and six attitude items as predictors: acceptance of receiving an AI diagnosis, belief that AI improves nutritional decision-making, reliance on AI as an initial plan before visiting a dietitian, belief that AI reduces visits for minor issues, belief that AI increases nutritional awareness, and the feeling that AI helps in understanding nutritional health information better than other sources.

Independent sociodemographic predictors of frequent AI use: An adjusted binary logistic regression was performed with frequent AI use (daily or several times per week) versus less frequent use (several times per month or once a month) as the outcome. The demographic variables that were significant in bivariate analysis (gender, age group, marital status, and chronic disease status) were entered simultaneously to control for confounding between them. Reference categories were male, age 21–30 years, married/divorced, and no chronic disease. Adjusted odds ratios (aOR) with 95% confidence intervals (CI) are reported, and model fit was assessed using the likelihood-ratio χ^2^ test and McFadden’s pseudo-R^2^.

Independent effects on nutritional practice: A multivariable linear regression was fitted with total practice score as the outcome. Gender, age group, marital status, education, region, income, health insurance, chronic disease, and BMI category were entered together with the standardized total attitude score, so that its coefficient represents the change in practice score per 1-SD increase in attitude score, adjusted for all other variables. Sparse categories were collapsed (education: high school or less, diploma/bachelor’s, postgraduate; region: Central versus other; income: <10,000, 10,000–19,999, ≥20,000 SAR). Unstandardized coefficients (β) with 95% CI, R^2^, and adjusted R^2^ are reported.

#### 2.3.6. Model Assumptions

For the linear regression models, multicollinearity was assessed using variance inflation factors (VIF; values below the conventional threshold of 5 considered acceptable), homoscedasticity using the Breusch–Pagan test, and normality of residuals using skewness, kurtosis, and the Shapiro–Wilk test. The total practice score is a continuous, approximately symmetric summated score, so it was modeled with linear regression rather than categorical or ordinal methods. Because the number of observations per predictor exceeded 10, modest departures from residual normality were not expected to affect inference [33]. For the model that showed evidence of heteroscedasticity, heteroscedasticity-consistent (HC3) robust standard errors were used for inference. Diagnostic results are reported in Section 3.

In the linear regression predicting total practice score from the six attitude items, all predictors had VIF values well below 5 (range 1.43–2.54), and the Breusch–Pagan test showed no significant heteroscedasticity (*p* = 0.411). Residuals showed negligible skewness (0.04) and modestly elevated kurtosis (0.79). The Shapiro–Wilk test detected a statistically significant but practically minor deviation from normality (*p* = 0.019), which was not considered problematic given the sample size [33]. The total practice score ranged from 17 to 62 (mean = 38.7, SD = 8.6). In the adjusted regression predicting total practice score from sociodemographic characteristics and standardized total attitude score, all predictors had acceptable VIF values (range 1.04–5.23). The Breusch–Pagan test indicated significant heteroscedasticity (*p* = 0.033); heteroscedasticity-consistent (HC3) robust standard errors were therefore used to compute *p*-values and confidence intervals for this model. Residuals showed minimal skewness (0.11) and kurtosis (0.28), and the Shapiro–Wilk test did not detect a significant deviation from normality (*p* = 0.256). Applying robust standard errors did not materially change the pattern of significance: the standardized total attitude score remained the only significant predictor of practice (β = 5.32, robust SE = 0.38, *p* < 0.001), while all sociodemographic predictors remained non-significant.

## 3. Results

This section presents the study results on the relationship between sociodemographic factors and adults’ use and reliance on AI for seeking nutritional health information in Saudi Arabia.

Table 2 demonstrates the demographic characteristics and health profile of the study participants. The sample consisted predominantly of Saudi nationals (96.84%) and females (75.53%). The most represented age group was 21–30 years (39.74%). In terms of marital status, 63.42% of participants were single. Most respondents lived in the Central region of Saudi Arabia (77.11%). Regarding educational attainment, 67.89% of participants held a bachelor’s degree. Household income levels varied, with the largest proportion of participants reporting a monthly income of less than 10,000 SAR (34.47%). From a health perspective, 61.58% of participants reported not having health insurance, while 77.63% reported no history of chronic diseases. Based on body mass index (BMI), the largest proportion of participants were classified as having normal weight (39.74%).

In Table 3, the results demonstrated that most participants reported using AI several times per week (43.16%). ChatGPT was the most commonly used tool (58.87%).

Among AI users, the most frequently reported pattern was using AI several times per week (43.16%), followed by daily use (33.42%). Significant associations were observed between frequency of AI use and gender (*p* < 0.001), age (*p* < 0.0001), and marital status (*p* < 0.001). Among chronic disease categories, significant associations were observed for hypertension (*p* = 0.0046), high cholesterol (*p* < 0.0001), asthma (*p* = 0.0491), thyroid disorder (*p* = 0.0066), kidney disease (*p* = 0.0331), and reporting none of the listed chronic conditions (*p* < 0.001). No significant associations were observed for nationality, educational level, region of residence, household income, health insurance status, BMI category, or diabetes. These findings are presented in Table 4.

The multivariable regression model predicting total nutritional practice was statistically significant (F = 45.36, *p* < 0.0001; adjusted R^2^ = 0.408). Significant positive associations were observed for acceptance of receiving an AI diagnosis (β = 1.426, *p* = 0.0002), belief that AI improves nutritional decision-making (β = 1.423, *p* = 0.0164), reliance on AI as an initial plan before visiting a dietitian (β = 0.877, *p* = 0.0458), belief that AI reduces visits for minor issues (β = 1.569, *p* = 0.0002), and belief that AI facilitates understanding of nutritional health information better than other sources (β = 1.003, *p* = 0.0415). The belief that AI increases nutritional awareness was not significantly associated with total practice (β = 0.460, *p* = 0.1341) (Table 5).

Table 6 presents the distribution of responses to perceived benefits of AI for nutritional habits, physical activity, and exercise planning. The highest proportion of agreement for exercise planning was observed among participants aged 21–30 years. A statistically significant association was observed between age and AI use for exercise planning (*p* = 0.0073). No significant associations with age were observed for nutritional habits (*p* = 0.4255) or physical activity (*p* = 0.3206).

Framed within trust-extended TAM, the regression model (Table 5) indicates that both Trust and Perceived Usefulness constructs independently predicted total practice scores. Acceptance of receiving an AI diagnosis—a Trust item—was among the strongest positive predictors of nutritional practice (β = 1.43, *p* = 0.0002), alongside the belief that AI reduces the need for dietitian visits for minor issues, a Behavioral Intention/Reliance item (β = 1.57, *p* = 0.0002). Perceived Usefulness beliefs also predicted practice, including the belief that AI improves nutritional decision-making (β = 1.42, *p* = 0.0164) and the perception that AI explains nutritional information more clearly than other sources (β = 1.00, *p* = 0.0415). Reliance on AI as an initial plan before visiting a dietitian, a further Behavioral Intention/Reliance item, was a weaker but still significant predictor (β = 0.88, *p* = 0.0458). Only the general Perceived Usefulness item on increased awareness (β = 0.46, *p* = 0.1341) failed to reach significance.

After mutual adjustment, age group was the only independent predictor of frequent AI use. Compared with respondents aged 21–30, those aged 41–50 (aOR = 0.24, 95% CI 0.07–0.85, *p* = 0.027) and 51–64 (aOR = 0.11, 95% CI 0.02–0.51, *p* = 0.005) had significantly lower adjusted odds of frequent use (Table 7). Gender, marital status, and chronic disease status, which were significantly associated with AI use in the unadjusted Chi-square analysis (Table 3), were no longer independently significant once age was controlled for, indicating that their bivariate associations were substantially confounded by age (Table 7).

In the fully adjusted model, total attitude score was by far the strongest predictor of nutritional practice: each 1-SD increase in attitude score was associated with a 5.32-point increase in total practice score (95% CI 4.63–6.01, *p* < 0.001). None of the sociodemographic variables—including those significant in bivariate analysis—remained independently significant predictors of practice after adjustment, with the exception of a borderline trend for female gender (β = 1.96, *p* = 0.061). The model explained 42.2% of the variance in practice scores (adjusted R^2^ = 0.395) (Table 8).

## 4. Discussion

### 4.1. Prevalence of Demographic and Health Characteristics

The results demonstrate a high prevalence of young, single, and educated Saudi females within the study sample, particularly those residing in the Central region. This dominant demographic profile, specifically the concentration of respondents in the 21–30 years age group, is a critical factor in the context of AI adoption. The prevalence of this younger cohort aligns with national trends showing that Saudi youth are the most frequent users of digital health tools, suggesting that age is a primary driver for the high engagement with AI for nutritional health information observed in this study. Furthermore, the widespread prevalence of bachelor’s degrees indicates a high baseline of digital literacy, allowing participants to effectively navigate complex AI interfaces.

From an economic perspective, there is a notable prevalence of participants reporting a monthly income of less than 10,000 SAR. While lower income is typically associated with poorer nutritional outcomes, it is significant that normal BMI remained the most prevalent weight status in this group. This is an encouraging finding that differs from national surveys reporting higher obesity rates [34]. The high prevalence of individuals reporting no history of chronic diseases further supports this interpretation of AI as a tool for primary prevention among this group.

### 4.2. Association of Sociodemographic Characteristics with AI Use

The current study found that female participants reported higher use of AI tools compared with males. However, this finding is inconsistent with previous research, which has reported higher levels of AI use among males. Although both studies included adult populations, the discrepancy in results may be explained by contextual and regional differences. Variations in cultural norms, digital infrastructure, and technology adoption patterns across different settings may influence gender-related differences in AI utilization and contribute to the observed inconsistency [35].

Participants aged 21–30 were the most frequent users of AI for nutritional information. This finding is consistent with previous research suggesting that younger individuals tend to be more comfortable using AI tools and demonstrate higher levels of digital confidence compared with older adults. Overall, this pattern indicates that age may play an important role in shaping engagement with AI-based health and nutrition resources, with younger populations being more likely to adopt and utilize these technologies [36].

Single participants reported higher use of AI tools compared with married participants. This finding is supported by previous research showing higher usage of ChatGPT among unmarried individuals compared with married individuals. This difference may be explained by greater flexibility and fewer family-related responsibilities among unmarried individuals, which can facilitate more frequent engagement with digital technologies [30].

Furthermore, the findings showed that participants without chronic diseases reported higher use of AI tools compared with those with chronic diseases. However, previous studies have not specifically compared AI use between individuals with and without chronic diseases. Therefore, this finding indicates a potentially important research gap in this area and highlights the need for further studies to examine how chronic disease status may influence the adoption and use of AI technologies.

### 4.3. Participants’ Reliance on AI for Nutritional Health Information

Our study found that participants were more open to the idea of receiving a diagnosis from AI. This was followed by their belief that AI could reduce the need to visit a nutritionist in simple cases, then their belief that AI could improve the ability to make healthy nutritional decisions and enhance understanding of nutritional information, and, finally, their willingness to rely on it as a preliminary plan before visiting a nutritionist. These results indicate that participants view AI as a useful tool for obtaining nutritional information, especially in situations that do not require complex intervention from a nutritionist.

This result is consistent with what a previous study indicated regarding the acceptance and reliance on AI in the field of health nutrition, which is related to the degree of trust in its accuracy, ease of use, and ability to provide quick and relevant information to the user [37]. Also, the higher acceptance of the idea of diagnosis from AI may reflect the willingness of participants to rely on it in basic tasks that require quick interpretation of information, which is consistent with the literature indicating that the public is often more receptive to AI when it is used as an aid and not as a complete replacement for a dietician [37].

Furthermore, the participants’ belief that AI might reduce their visits to nutritionists for minor health issues can be explained by their desire for quick access to answers without needing direct consultation with a specialist, especially for common or uncomplicated nutritional matters. This aligns with the findings of a recent study indicating that AI is increasingly viewed as a first step toward obtaining health advice or guidance, particularly for simple and specific problems [38].

As for the participants’ belief that AI improves nutritional health decision-making, this indicates that users view AI as a supportive tool that can help them compare food options and better understand recommendations. Previous studies support the idea that AI provides readily available information and combines diverse data in a way that can facilitate an individual’s initial understanding of the appropriate decision [38] and have also suggested that AI can be beneficial in nutritional education and providing personalized information that contributes to improved health awareness [31].

The results also showed that participants believe AI helps them understand healthy nutrition information better than any other source, highlighting the importance of accessibility and clarity in the content it provides. This may be because some users find that AI tools offer simplified and straightforward explanations, making them more appealing than other sources. This aligns with recent studies indicating that AI can enhance users’ understanding of healthy nutrition when information is presented in a way that is tailored to their needs and clearly explained [31,38].

Regarding the use of AI as an initial step before consulting a nutritionist, this item received a relatively lower rating compared with the other items. This may indicate that participants still prefer reliable, direct support when transitioning from information gathering to treatment. This result can be explained by the fact that AI is perceived as a useful tool for initial exploration, but it remains less reliable than a professional in cases requiring advanced skills, careful assessment, or personal intervention [37]. This suggests that AI in the field of nutritional health is often used as a supplementary tool, not as a complete replacement for a nutritionist [37].

### 4.4. Age Factor and the Use of AI in Exercise Planning

The results of the current study showed a statistically significant correlation between young age (21–30 years old) and the use of AI in planning a sports schedule. In contrast, the results did not show any statistically significant association between the use of AI and a change in dietary habits or the level of physical activity. The high use of AI in the planning of exercises in the younger category can be explained by the fact that they are better able to cope with modern technologies. Previous studies have indicated that younger age groups are the most used for health applications [39], but these studies focused on general use, especially in the field of nutrition, and did not address the use of AI in Exercise Planning, which makes the results of this study different in this aspect. As for older groups, they may face challenges in using these technologies, such as difficulty handling user interfaces or a low level of confidence in their accuracy. The literature indicated lower performance expectations and less positive attitudes towards technology in older adults [40], without directly addressing Exercise Planning behavior. Based on the researcher’s review of the available literature, no studies have been found that explicitly addressed this relationship, so no studies have been found that support or disagree with this particular finding, indicating a research gap in this area.

The multivariable logistic regression provides an important refinement of the bivariate findings by showing that age was the only sociodemographic characteristic independently associated with frequent AI use after simultaneous adjustment for gender, marital status, and chronic disease status. Although gender and marital status were significantly associated with AI-use frequency in the bivariate analysis, these associations were no longer statistically significant after adjustment, suggesting that their initial relationships may have been partly explained by differences in age distribution within the sample. This distinction between unadjusted and adjusted associations is particularly relevant when interpreting technology-use patterns, as demographic characteristics may operate as external factors that shape exposure to, familiarity with, and acceptance of emerging technologies rather than directly determining use. This interpretation is consistent with the TAM and subsequent health-information-system research, in which demographic characteristics are generally considered external variables, whereas technology-related perceptions such as perceived usefulness and ease of use are more proximal to technology acceptance and use [41]. In the Saudi context, the observed age gradient is also consistent with evidence from a large national study showing significant age-related differences in digital health literacy, with younger adults generally demonstrating greater proficiency in obtaining and evaluating health information online [16]. Nevertheless, because the present study is cross-sectional, age should not be interpreted as a causal determinant of frequent AI use; it may instead reflect differences in digital health literacy, previous exposure to generative AI, or other unmeasured characteristics.

In the multivariable model of nutritional practice, attitude toward AI remained strongly and independently associated with practice, whereas the sociodemographic characteristics included in the model were no longer statistically significant (adjusted β = 5.32, 95% CI: 4.63–6.01, *p* < 0.001). This pattern is broadly consistent with the central logic of TAM, whereby users’ perceptions and evaluations of a technology are expected to be more proximal to behavioral outcomes than demographic characteristics [41]. It is also consistent with a broader systematic review of AI acceptance, which identified perceived usefulness, attitudes, trust, and effort expectancy among the factors positively associated with intention, willingness, and use across different AI applications [23]. The item-level findings in the present study further suggest that perceptions related to the usefulness of AI for nutritional decision-making and understanding, as well as trust and willingness to rely on AI, may be particularly relevant to reported nutritional practices. However, these findings should be interpreted cautiously because the present study did not use a fully validated TAM instrument, and the mapping of questionnaire items onto TAM constructs was performed post hoc. Moreover, positive attitudes toward AI should not be equated with the accuracy or safety of AI-generated nutritional information. Evidence from nutritional chatbot evaluations demonstrates substantial variability in accuracy, completeness, reproducibility, and consistency, particularly in complex clinical scenarios [9]. Similarly, research on trust in healthcare AI emphasizes the importance of achieving appropriate rather than unconditional trust, since excessive trust in erroneous AI outputs may be as problematic as unjustified distrust [42]. Thus, the present findings suggest that future efforts to promote the appropriate use of AI for nutritional health information should consider attitudinal factors such as perceived usefulness and calibrated trust, while simultaneously strengthening digital health literacy and users’ ability to critically evaluate AI-generated information and recognize when professional nutrition guidance is required.

### 4.5. Limitations

This study has several limitations that should be considered when interpreting the findings. First, the cross-sectional design captures AI use, attitudes, and practices at a single point in time and therefore cannot establish causality or temporal sequence between sociodemographic characteristics, attitudes, and reliance on AI for nutritional health information [43,44]. The associations reported, including the regression findings, should be interpreted as evidence of association rather than causal effect. Longitudinal or prospective designs are needed to determine whether attitudes toward AI precede, and causally drive, changes in nutritional practice over time. Second, all data were self-reported via an online questionnaire, which is subject to social desirability bias and recall bias. Participants may have overstated healthy or socially favorable behaviors [45] (e.g., consulting a dietitian) or understated reliance on AI tools perceived as less legitimate sources of health information, and frequency-of-use items depended on participants’ ability to accurately recall their own usage patterns. No objective or app-based usage data were available to corroborate self-reports. Third, the convenience sampling method—recruitment via social media and in-person distribution at a single, female-only university (Princess Nourah bint Abdulrahman University) and Riyadh-based public venues—produced a sample that is not representative of the general Saudi adult population. As detailed above, the sample substantially overrepresented women (75.97% vs. an approximately balanced national gender distribution), single individuals (64.08%), university-educated respondents (67.70% vs. ~36% nationally), and Central-region residents (77.26% vs. ~27% nationally) (General Authority for Statistics, 2023; World Data, 2024). This self-selection is typical of online convenience surveys and limits the generalizability of the findings to the broader [46,47], more demographically diverse adult population of Saudi Arabia; results should be interpreted as reflecting this specific subgroup rather than the national population. Fourth, the trust-extended TAM framework used to interpret the findings was applied post hoc to an already-collected, previously content-validated instrument rather than used prospectively to design it, and constructs corresponding to Perceived Ease of Use, Effort Expectancy, and Social Influence were not measured and could not be assessed. Taken together, these limitations suggest that the findings are best understood as an exploratory, descriptive account of AI reliance among a specific, digitally engaged subgroup of Saudi adults, providing a foundation for future studies using probability-based sampling, longitudinal designs, and objective usage measures to confirm and extend these results.

## 5. Conclusions

This cross-sectional study found a high prevalence of AI use for nutritional health information among a predominantly young, female, single, Central-region, and highly educated sample of Saudi adults. Although age, gender, and marital status were associated with AI-use frequency in bivariate analyses, age was the only independent sociodemographic predictor of frequent AI use after adjustment. In contrast, attitudes toward AI were the strongest independent predictor of nutritional practice, highlighting the importance of trust and perceived usefulness in shaping AI-related behaviors. A key strength of this study is the use of multivariable analyses to distinguish independent demographic associations from confounding and to examine the contribution of attitudes to nutritional practices. The study also provides an initial application of a trust-extended Technology Acceptance Model to AI use for nutritional health information in the Saudi context. However, several factors limit causal inference, representativeness, and generalizability. Future research should incorporate a fully specified technology-acceptance framework, including perceived ease of use, social influence, and facilitating conditions, and should investigate how digital health literacy, trust, and the accuracy and cultural appropriateness of AI-generated nutritional information influence its safe and effective use. These approaches can help inform evidence-based strategies for integrating AI into nutrition and digital health services while maintaining appropriate professional oversight.

## Figures and Tables

**Table 1:** Mapping of questionnaire content to trust-extended TAM constructs.

TAM Construct	Questionnaire Content	Role in Analysis
External variables	Section 1 (sociodemographic and health characteristics)	Predictors acting indirectly through beliefs
Trust	Items 16, 17, 26	Belief construct
Perceived usefulness	Items 19, 20, 22, 25, 27–29	Belief construct
Behavioral intention/reliance	Items 15, 18, 21, 23, 24	Belief construct
Use behavior	Items 30–43 (practice section)	Outcome (total practice score)
AI usage patterns	Section 2	Prevalence, frequency, and platforms of use
Perceived ease of use	Not measured	Acknowledged as a limitation

TAM: Technology Acceptance Model.

**Table 2:** Demographics and Health Profiles of AI users (n = 380).

Variable	N	% of Total
Nationality		
Saudi	368	96.84
Non-Saudi	12	3.16
Gender		
Female	287	75.53
Male	93	24.47
Age		
18–20 years	98	25.79
21–30 years	151	39.74
31–40 years	40	10.53
41–50 years	73	19.21
51–64 years	18	4.74
Current marital status		
Unmarried	241	63.42
Married	139	36.58
Region of Residence (Saudi Arabia)		
Southern	8	2.11
Eastern	18	4.74
Northern	8	2.11
Western	53	13.95
Central	293	77.11
Educational level		
High school or less	63	16.58
Diploma	22	5.79
Bachelor`s degree	258	67.89
Postgraduate degree	37	9.74
Household Monthly Income		
Less than 10,000 SAR	131	34.47
10,000–19,999 SAR	113	29.74
20,000–29,999 SAR	67	17.63
30,000 SAR or more	69	18.16
Health Insurance Status		
Yes, I do have health insurance	146	38.42
No, I do not have health insurance	234	61.58
Chronic Disease Status (Multiple Selection Allowed)		
High cholesterol	31	8.16
Hypertension	15	3.95
Thyroid disorder	21	5.53
Asthma	32	8.42
Diabetes	22	5.79
None of the above	3	0.79
BMI categorization		
Normal weight	151	39.74
Obesity	107	28.16
Overweight	79	20.79
Underweight	43	11.32

**Table 3:** AI Usage Patterns for Nutritional Information (n = 380).

Variable	N	% of Total
Frequency of Artificial Intelligence Tool Usage		
Daily	127	33.42
Several times a week	164	43.16
Several times a month	60	15.79
Once a month	29	7.63
Artificial Intelligence Tools Used for Nutritional Health Information *		
Adobe AI	9	2.37
ChatGPT	358	94.21
Claude	8	2.11
DeepSeek	30	7.89
Google Gemini	178	46.84
Microsoft Copilot	24	6.32

* The AI tool counts are based on each tool being selected at least once. Multiple tool selection was allowed.

**Table 4:** Association Between AI Usage and Sociodemographic Characteristics (n = 380).

Frequency of AI Use	Daily Total N (%)	Several Times a Week Total N (%)	Several Times a Month Total N (%)	Once a Month Total N (%)	*p*-Value	
Saudi Nationality:						
Saudi	123 (32.37)	159 (41.84)	58 (15.26)	28 (7.37)	0.9981	Fisher
Non-Saudi	4 (1.05)	5 (1.32)	2 (0.53)	1 (0.26)		
Gender:						
Female	106 (27.89)	127 (33.42)	40 (10.53)	14 (3.68)	0.0001 *	χ^2^
Male	21 (5.53)	37 (9.74)	20 (5.26)	15 (3.95)		
Age:						
18–20 years	44 (11.58)	42 (11.05)	10 (2.63)	2 (0.53)	<0.0001 *	Fisher
21–30 years	56 (14.74)	74 (19.47)	18 (4.74)	3 (0.79)		
31–40 years	6 (1.58)	22 (5.79)	7 (1.84)	5 (1.32)		
41–50 years	19 (5.00)	22 (5.79)	17 (4.47)	15 (3.95)		
51–64 years	2 (0.53)	4 (1.05)	8 (2.11)	4 (1.05)		
Current Marital Status:						
Unmarried	97 (25.53)	111 (29.21)	27 (7.11)	6 (1.58)	<0.0001 *	χ^2^
Married	30 (7.89)	53 (13.95)	33 (8.68)	23 (6.05)		
Educational Level:						
High school or less	25 (6.58)	18 (4.74)	12 (3.16)	8 (2.11)	0.2350	Fisher
Diploma	6 (1.58)	11 (2.89)	3 (0.79)	2 (0.53)		
Bachelor’s degree	80 (21.05)	123 (32.37)	40 (10.53)	15 (3.95)		
Postgraduate degree	16 (4.21)	12 (3.16)	5 (1.32)	4 (1.05)		
Region of Residence:						
Central	104 (27.37)	123 (32.37)	42 (11.05)	24 (6.32)	0.1878	Fisher
Western	14 (3.68)	24 (6.32)	14 (3.68)	1 (0.26)		
Southern	2 (0.53)	6 (1.58)	0 (0.00)	0 (0.00)		
Eastern	4 (1.05)	8 (2.11)	3 (0.79)	3 (0.79)		
Northern	3 (0.79)	3 (0.79)	1 (0.26)	1 (0.26)		
Monthly Household Income:						
Less than 10,000 SAR	44 (11.58)	64 (16.84)	18 (4.74)	5 (1.32)	0.1657	Fisher
10,000–19,999 SAR	36 (9.47)	51 (13.42)	18 (4.74)	8 (2.11)		
20,000–29,999 SAR	21 (5.53)	29 (7.63)	10 (2.63)	7 (1.84)		
30,000 SAR or more	12 (3.16)	12 (3.16)	9 (2.37)	7 (1.84)		
Health Insurance:						
Yes, I have health insurance	49 (12.89)	65 (17.11)	22 (5.79)	10 (2.63)	0.9452	χ^2^
No, I do not have health insurance	78 (20.53)	99 (26.05)	38 (10.00)	19 (5.00)		
BMI Categorization:						
Normal weight	55 (14.47)	68 (17.89)	22 (5.79)	9 (2.37)	0.2576	Fisher
Obesity	32 (8.42)	47 (12.37)	19 (5.00)	9 (2.37)		
Overweight	21 (5.53)	32 (8.42)	16 (4.21)	10 (2.63)		
Underweight	19 (5.00)	17 (4.47)	3 (0.79)	1 (0.26)		
Chronic Diseases (Multiple Selection Allowed):						
Diabetes	11 (2.89)	6 (1.58)	3 (0.79)	2 (0.53)	0.2905	Fisher
Hypertension	4 (1.05)	2 (0.53)	5 (1.32)	4 (1.05)	0.0046 *	Fisher
High cholesterol	9 (2.37)	5 (1.32)	7 (1.84)	10 (2.63)	<0.0001 *	Fisher
Asthma	14 (3.68)	7 (1.84)	8 (2.11)	3 (0.79)	0.0491 *	Fisher
Thyroid disorder	3 (0.79)	9 (2.37)	3 (0.79)	6 (1.58)	0.0066 *	Fisher
Heart disease	1 (0.26)	1 (0.26)	1 (0.26)	0 (0.00)	0.6723	Fisher
Kidney disease	0 (0.00)	0 (0.00)	1 (0.26)	1 (0.26)	0.0331	Fisher
None of the above	98 (25.79)	141 (37.11)	42 (11.05)	14 (3.68)	<0.001 *	χ^2^

* Percentages are based on the total AI-user sample (N = 380). Fisher’s exact test was used when one or more expected cell counts were <5; otherwise, Pearson’s chi-square test was used. For chronic disease status, multiple responses were permitted; therefore, categories are not mutually exclusive and percentages do not sum to 100%.

**Table 5:** Impact of AI Reliance on Nutritional Practices.

Term	Estimate β	Std Error	t Ratio	Prob > |*p*|
Intercept	−0.084813	1.701252	−0.05	0.9603
Acceptance of receiving AI diagnosis	1.4259058	0.381323	3.74	0.0002 *
Belief that AI improves nutritional decision-making	1.4225112	0.590196	2.41	0.0164 *
Reliance on AI as an initial plan before visiting a dietitian	0.8765784	0.437392	2.00	0.0458 *
Belief that AI reduces visits for minor issues	1.5691246	0.419747	3.74	0.0002 *
Belief that AI increases awareness	0.460141	0.306473	1.50	0.1341
Feeling that AI helps understand nutritional health information better than other sources	1.0033439	0.4906	2.05	0.0415 *

* Note: A *p*-value < 0.05 was considered statistically significant.

**Table 6:** Age vs. Perceived Benefits of AI for Lifestyle.

Variable						*p*-Value (Chi-Square)
Age	18–20 Years Total N (%)	21–30 Years Total N (%)	31–40 Years Total N (%)	41–50 Years Total N (%)	51–64 Years Total N (%)	
Artificial intelligence (AI) for nutritional habits.						
Strongly agree	21 (5.53)	32 (8.42)	7 (1.84)	8 (2.11)	1 (0.26)	0.4255
Agree	47 (12.37)	80 (21.05)	24 (6.32)	40 (10.53)	10 (2.63)	
Neutral	22 (5.79)	32 (8.42)	6 (1.58)	21 (5.53)	7 (1.84)	
Disagree	6 (1.58)	7 (1.84)	3 (0.79)	3 (0.79)	0 (0.00)	
Strongly disagree	2 (0.53)	0 (0.00)	0 (0.00)	1 (0.26)	0 (0.00)	
Artificial intelligence (AI) for physical activity.						
Strongly agree	22 (5.79)	38 (10.00)	8 (2.11)	9 (2.37)	2 (0.53)	0.3206
Agree	43 (11.32)	70 (18.42)	22 (5.79)	45 (11.84)	11 (2.89)	
Neutral	21 (5.53)	33 (8.68)	8 (2.11)	15 (3.95)	4 (1.05)	
Disagree	9 (2.37)	10 (2.63)	2 (0.53)	2 (0.53)	1 (0.26)	
Strongly disagree	3 (0.79)	0 (0.00)	0 (0.00)	2 (0.53)	0 (0.00)	
Artificial intelligence (AI) for exercise planning.						
Strongly agree	33 (8.68)	48 (12.63)	11 (2.89)	9 (2.37)	2 (0.53)	0.0073
Agree	41 (10.79)	73 (19.21)	25 (6.58)	52 (13.68)	12 (3.16)	
Neutral	18 (4.74)	27 (7.11)	3 (0.79)	8 (2.11)	4 (1.05)	
Disagree	4 (1.05)	3 (0.79)	1 (0.26)	3 (0.79)	0 (0.00)	
Strongly disagree	2 (0.53)	0 (0.00)	0 (0.00)	1 (0.26)	0 (0.00)	

*p*-values computed via Fisher’s exact test was used when one or more expected cell counts were <5.

**Table 7:** Adjusted Binary Logistic Regression Predicting Frequent AI Use.

Predictor (Ref. Category)	OR	95% CI	*p*
Gender: Female (ref. Male)	0.97	0.49–1.94	0.930
Age 18–20 (refs. [21,22,23,24,25,26,27,28,29,30])	1.12	0.52–2.41	0.774
Age 31–40 (refs. [21,22,23,24,25,26,27,28,29,30])	0.40	0.12–1.42	0.157
Age 41–50 (refs. [21,22,23,24,25,26,27,28,29,30])	0.24	0.07–0.85	0.027
Age 51–64 (refs. [21,22,23,24,25,26,27,28,29,30])	0.11	0.02–0.51	0.005
Marital status: Unmarried (ref. Married)	1.07	0.34–3.40	0.907
Chronic disease: Yes (ref. No)	0.64	0.35–1.17	0.147

Multivariable logistic regression of frequent AI use (daily or several times/week, n = 291) versus less frequent use (several times/month or once a month, n = 89) on sociodemographic variables found significant in bivariate analysis, n = 380. Model: N = 380; χ^2^(7) = 49.3, *p* < 0.001; McFadden’s pseudo-R^2^ = 0.119.

**Table 8:** Multivariable Linear Regression Predicting Total Practice Score.

Predictor (Ref. Category)	β (Unstd.)	95% CI	*p*
Gender: Female (ref. Male)	1.96	−0.09–4.01	0.061
Age 18–20 (refs. [21,22,23,24,25,26,27,28,29,30])	−0.27	−2.04–1.50	0.764
Age 31–40 (refs. [21,22,23,24,25,26,27,28,29,30])	−1.28	−4.82–2.27	0.480
Age 41–50 (refs. [21,22,23,24,25,26,27,28,29,30])	−1.15	−4.80–2.49	0.534
Age 51–64 (refs. [21,22,23,24,25,26,27,28,29,30])	−1.85	−6.43–2.72	0.426
Marital status: Unmarried (ref. Married)	0.09	−3.06–3.23	0.957
Education: Diploma/Bachelor’s (ref. High school or less)	0.10	−1.81–2.02	0.915
Education: Postgraduate (ref. High school or less)	−1.01	−4.09–2.07	0.520
Region: Other (ref. Central)	0.19	−1.49–1.87	0.823
Income 10,000–19,999 SAR (ref. <10,000 SAR)	0.56	−1.20–2.32	0.534
Income ≥20,000 SAR (ref. <10,000 SAR)	−0.63	−2.39–1.14	0.486
Health insurance: Yes (ref. No)	−0.84	−2.28–0.59	0.249
Chronic disease: Yes (ref. No)	−0.60	−2.37–1.16	0.502
BMI: Underweight (ref. Normal)	0.87	−1.46–3.21	0.461
BMI: Overweight (ref. Normal)	0.10	−1.67–1.86	0.915
BMI: Obese (ref. Normal)	−0.94	−3.00–1.12	0.371
**Total Attitude score (per 1-SD increase)**	**5.32**	**4.63–6.01**	**<0.001**

Adjusted linear regression of total nutritional-practice score on sociodemographic variables and total attitude score (standardized), n = 380. Model: N = 380; F(17, 362) = 15.56, *p* < 0.001; R^2^ = 0.422, Adjusted R^2^ = 0.395. Given evidence of heteroscedasticity (Breusch–Pagan *p* = 0.033), coefficient standard errors, *p*-values, and 95% confidence intervals were computed using heteroscedasticity-consistent (HC3) robust standard errors.

## Data Availability

The data presented in this study are available on request from the corresponding author. The data are not publicly available due to privacy and ethical restrictions, as the dataset contains potentially identifiable participant information.

## Supplementary Materials

The following supporting information can be downloaded at: https://www.mdpi.com/article/10.3390/healthcare14213702/s1. File S1: Questionnaire.

## Abbreviations

The following abbreviations are used in this manuscript:

AI	Artificial Intelligence
CITC	Communications and Information Technology Commission
MOH	Ministry of Health
X-ray	X-radiation
ChatGPT	Chat Generative Pre-Trained Transformer
BMI	Body Mass Index
TAM	Technology Acceptance Model
ML	Machine Learning
DL	Deep Learning
USDA	United States Department of Agriculture
PNU	Princess Nourah bint Abdulrahman University
HISB	Online Health Information Seeking Behavior
SVM	Support Vector Machines
CNNs	Convolutional Neural Networks
Bard AI	Gemini
Bing Chat	Microsoft Copilot
