# Supplementary material for: The Impact of Demographic Factors on Adults’ Use of Artificial Intelligence for Seeking Nutritional Health Information in Saudi Arabia
Source: Healthcare (Basel). 2026 Nov 1;14(21):3702. doi: 10.3390/healthcare14213702 (PMC13762811; doi:10.3390/healthcare14213702)
Supplement: Supplementary file 1 [file healthcare-14-03702-s001.zip › healthcare-4513267-supplementary.pdf]

Supplementary File S1: Questionnaire

Do you agree to participate in the survey?
- Yes
- No

Do you use artificial intelligence?
- Yes
- No

Demographic Questions:

1. Are you a Saudi national?

- Yes
- No

2. Gender:

- Female
- Male

3. Age:

- Under 18 years
- 18–20 years
- 21–30 years
- 31–40 years
- 41–50 years
- 51–64 years
- 65 years or older

4. Current marital status:

- Single
- Married
- Divorced
- Widowed

5. Educational level:

- High school or less
- Bachelor’s degree
- Postgraduate degree

6. Which region of Saudi Arabia do you currently live in?

- Central
- Western
- Southern
- Eastern
- Northern

7. What is your household’s monthly income?

• Less than 10,000 SAR
• 10,000–19,999 SAR
• 20,000–29,999 SAR
• 30,000–39,999 SAR
• 40,000–49,999 SAR
• 50,000 SAR or more

8. Do you currently have health insurance?

- Yes, I have health insurance
- No, I do not have health insurance

9. What is your current height in centimeters?

Height: __ cm

10. What is your current weight in kilograms?

Weight: __ kg

11. Do you have any chronic diseases?

- ☐ Diabetes
- ☐ Hypertension
- ☐ High cholesterol
- ☐ Asthma
- ☐ Heart disease
- ☐ Kidney disease
- ☐ Thyroid disorder
- ☐ None of the above

Uses of artificial intelligence:

12. How often do you use artificial intelligence tools?

- Daily
- Several times a week
- Several times a month
- Once a month
- I did not use AI

13. Which artificial intelligence tools do you use for health nutrition information?

- ChatGPT
- Google Gemini
- Microsoft Copilot
- Claude
- DeepSeek
- Adobe AI

14. For which of the following nutrition-related purposes do you use artificial intelligence?

- ☐Weight management
- ☐Healthy diet planning
- ☐Calorie and nutrient tracking
- ☐Enhancing physical activity
- ☐Information about vitamins and supplements
- ☐Managing health conditions through diet (e.g., diabetes, hypertension)
- ☐Recipes and meal ideas
- ☐None

15. I believe that I prefer using AI tools rather than online search platforms for health or nutrition information.

- Strongly agree
- Agree
- Neutral
- Disagree
- Strongly disagree

Attitude:

16. I believe that artificial intelligence tools provide accurate and reliable nutritional health information.

- Strongly agree
- Agree
- Neutral
- Disagree
- Strongly disagree

17. I believe I would feel comfortable accepting a diagnosis from medical artificial intelligence tools.

- Strongly agree
- Agree
- Neutral
- Disagree
- Strongly disagree

18. I believe that I will rely on artificial intelligence tools to improve my health nutrition awareness in the future.

- Strongly agree
- Agree
- Neutral
- Disagree
- Strongly disagree

19. I think that using artificial intelligence contributes to increasing my nutritional health awareness.

- Strongly agree
- Agree
- Neutral
- Disagree
- Strongly disagree

20. I feel that artificial intelligence helps me understand nutritional health information better than other sources.

- Strongly agree
- Agree
- Neutral
- Disagree
- Strongly disagree

21. I think using artificial intelligence reduces my need to visit a dietitian for minor issues (Nutritional information such as preparing a dietary plan).

- Strongly agree
- Agree

- Neutral
- Disagree
- Strongly disagree

22. I believe using artificial intelligence enhances my ability to make better nutritional health decisions.

- Strongly agree
- Agree
- Neutral
- Disagree
- Strongly disagree

23. I can rely on artificial intelligence as an initial plan before visiting a Dietitian.

- Strongly agree
- Agree
- Neutral
- Disagree
- Strongly disagree

24. I believe artificial intelligence will play a bigger role in the future as a source of nutritional health information.

- Strongly agree
- Agree
- Neutral
- Disagree
- Strongly disagree

25. I believe that using artificial intelligence tools for nutritional health information can help reduce my financial burden.

- Strongly agree
- Agree
- Neutral
- Disagree
- Strongly disagree

26. Following AI-generated diet plans without consulting a nutritionist can lead to health risks.

- Strongly agree
- Agree
- Neutral
- Disagree
- Strongly disagree

27. I believe that using artificial intelligence can help improve my nutritional habits.

- Strongly agree
- Agree
- Neutral
- Disagree
- Strongly disagree

28. I believe that using artificial intelligence can help improve my physical activity habits.

- Strongly agree
- Agree
- Neutral
- Disagree
- Strongly disagree

29. Artificial intelligence (AI) can be a helpful tool in planning and organizing my exercise routine.

- Strongly agree
- Agree
- Neutral
- Disagree
- Strongly disagree

Practice:

30. When you experience a problem that you believe is related to your diet, do you seek help from artificial intelligence tools such as ChatGPT?

- Always
- Most of the time
- Sometimes
- Rarely
- Never

31. When you experience a problem that you believe is related to your diet, do you consult a nutritionist?

- Always
- Most of the time
- Sometimes
- Rarely
- Never

32. When you experience a problem that you believe is related to your diet, do you consult your family or friends?

- Always

- Most of the time
- Sometimes
- Rarely
- Never

33. Have you ever used artificial intelligence to self-diagnose before visiting a nutritionist?

- Always
- Most of the time
- Sometimes
- Rarely
- Never

34. Have you ever changed your diet based on information you got from artificial intelligence?

- Always
- Most of the time
- Sometimes
- Rarely
- Never

35. When the information from artificial intelligence conflicts with the advice of a nutritionist, I follow the nutritionist's advice.

- Always
- Most of the time
- Sometimes
- Rarely
- Never

36. When the information from artificial intelligence conflicts with the advice of a nutritionist, I follow the artificial intelligence's advice.

- Always
- Most of the time
- Sometimes
- Rarely
- Never

37. When the information from artificial intelligence conflicts with the advice of a nutritionist, I follow the advice of family or friends.

- Always
- Most of the time
- Sometimes
- Rarely
- Never

38. When the information from artificial intelligence conflicts with the advice of a nutritionist, I try to reconcile them.

- Always
- Most of the time
- Sometimes
- Rarely
- Never

39. Have you ever applied a nutrition plan you got from AI tools?

- Always
- Most of the time
- Sometimes
- Rarely
- Never

40. Have you ever followed an exercise routine recommended by AI tools?

- Always
- Most of the time
- Sometimes
- Rarely
- Never

41. Have you ever tried home remedies or recipes suggested by AI tools?

- Always
- Most of the time
- Sometimes
- Rarely
- Never

42. Have you ever followed medical advice or recommendations found through AI tools?

- Always
- Most of the time
- Sometimes
- Rarely
- Never

43. Have you never applied any information you got from AI?

- Always
- Most of the time
- Sometimes
- Rarely
- Never

This is the end of the questions

Thank you for participating
